# Supplementary material for: The causal relationship between the human gut microbiota and pyogenic arthritis: a Mendelian randomization study
Source: Front Cell Infect Microbiol. 2024 Nov 26;14:1452480. doi: 10.3389/fcimb.2024.1452480 (PMC11629706; doi:10.3389/fcimb.2024.1452480)
Supplement: Supplementary file 1 [file DataSheet1.zip › mendelian test/ebi-a-GCST90027770.csv_forest.pdf]

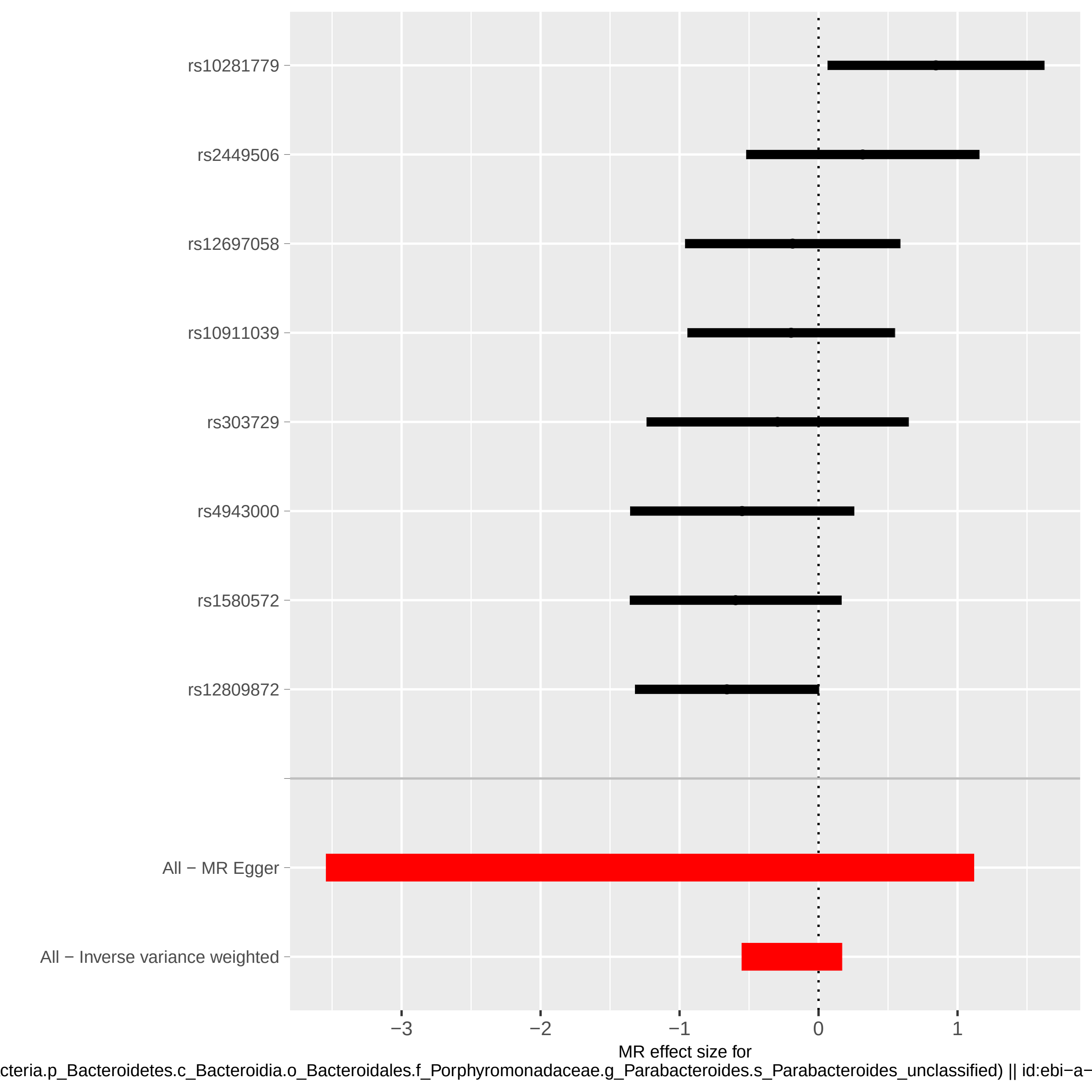
rs10281779
rs2449506
rs12697058
rs10911039
rs303729
rs4943000
rs1580572
rs12809872
All − MR Egger
All − Inverse variance weighted
−3
−2
−1
0
1
MR effect size for
cteria.p_Bacteroidetes.c_Bacteroidia.o_Bacteroidales.f_Porphyromonadaceae.g_Parabacteroides.s_Parabacteroides_unclassified) || id:ebi−a−
